# Patient-Centered Care in Psoriatic Arthritis—A Perspective on Inflammation, Disease Activity, and Psychosocial Factors

**DOI:** 10.3390/jcm9103103

**Published:** 2020-09-25

**Authors:** Bogdan Batko

**Affiliations:** Department of Rheumatology and Immunology, Faculty of Medicine and Health Sciences, Andrzej Frycz Modrzewski University, 30-705 Krakow, Poland; bpbatko@gmail.com

**Keywords:** depression, psoriatic arthritis, cognitive behavioral therapy, inflammation, adherence, multidisciplinary, psoriasis

## Abstract

Psoriatic arthritis (PsA) is a seronegative spondyloarthropathy characterized by skin lesions, dactylitis, and enthesitis. Patients with PsA suffer from a variety of psychosocial difficulties and nonspecific symptoms early on in the disease course and continue to experience progressive disease due to delays in diagnosis and treatment. Symptoms initially viewed as somatization could lead to undertreatment and promote psychological distress, poor coping, and negative patient–provider relationships. Pain and fatigue are important complaints that affect the patient’s perception and may need to be addressed with a multidisciplinary approach. Maladaptive cognitive responses can lead to a negative illness perception and impact patient beliefs and concerns over treatment, as well as nonadherence. An underlying inflammatory component in affective disorders has been examined, though whether and how it may interact mechanistically in PsA warrants interest. Cognitive behavioral therapy represents a nonpharmacological treatment modality that can be combined with cytokine-targeted therapy to address both somatic and psychological complaints. Future directions for research include: (1) Elucidating nonspecific manifestations (e.g., subclinical stage, differential with functional syndromes) of PsA and how they impact diagnosis and management; (2) characterizing immune-mediated components of mood disorders in PsA; and (3) whether a bidirectional approach with abrogating inflammation and psychotherapeutic support leads to improved outcomes.

## 1. Introduction

**Figure 1:**
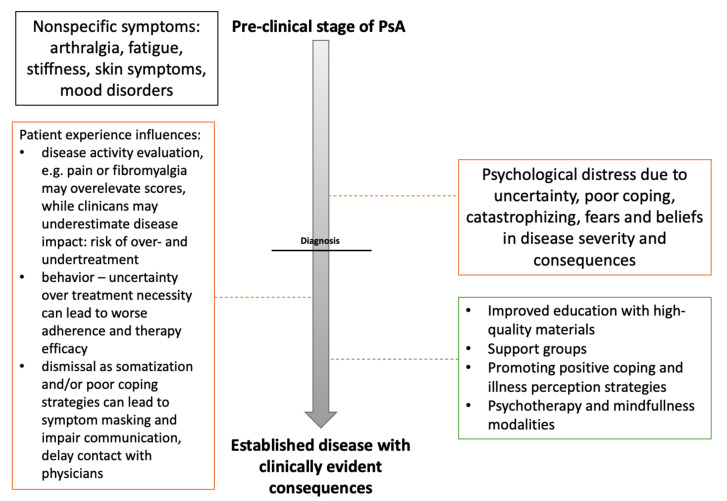
Proposed timeline schematic for psoriatic arthritis. Patients with psoriatic arthritis (PsA) may initially present with non-specific musculoskeletal symptoms that remain unclassified. Misdiagnoses as somatization can occur contributing to delays in diagnosis. Progression of untreated disease in patients with unfavorable coping strategies leads to increasing somatic and psychological distress, which shares a bidirectional link with poor treatment outcomes. Negative consequences are outlined in orange with a focus on management-related dimensions that can, potentially, be modified (dashed line; treatment options—green).

Psoriatic arthritis (PsA) is a seronegative spondyloarthropathy marked by skin disease, enthesitis, dactylitis, ankylosis, and uveitis. It affects nearly 20% of patients with psoriasis, and up to 25% with the presence of moderate-to-severe disease [1]. The immune pathogenesis of spondyloarthropathies, including PsA, relates to underlying inflammation driven by the pro-inflammatory TNFα and IL-23/IL-17 axes, which is coupled with underlying genetic predisposition [2]. PsA is a heterogenous disease: Several extraarticular features (cardiometabolic or gastrointestinal involvement) are well known, while psychological conditions and general symptoms (pain, fatigue) are being recognized as significant factors that impact assessments and quality of life (QoL). Expert groups have emphasized these aspects when devising management guidelines [3]. PsA is preceded by a preclinical phase with nonspecific musculoskeletal symptoms (e.g., pain, stiffness, joint tenderness), though not all patients with psoriasis will develop PsA, nor is family history of disease a deciding factor. Delays in the route to PsA diagnosis can commonly occur over several months [4], which can lead to mental distress and patient dissatisfaction (see Figure 1). Symptoms related to arthralgia (pain, stiffness, tenderness) or evidence of subclinical synovitis may confer a higher risk for developing PsA [5,6,7]. The socioeconomic burden of disease is also substantial with high healthcare costs and comorbidity, reduced work ability, and lower pay that may be present months prior to diagnosis [8,9].

## 2. Psychosocial Aspects and Quality of Life in Psoriatic Arthritis

The nature of PsA symptoms at early stages can often be misattributed to psychological distress rather than somatic illness, which leads to clinical inertia and progression of untreated disease, further exacerbating concerns over somatic manifestations. It has been observed that misdiagnoses, of which psychosomatic disorders represent over one fourth, are associated with delays in diagnosis [4]. Nontreatment and undertreatment have been reported for a large proportion of patients, with patient dissatisfaction over therapy remaining prevalent, suggesting that important patient domains are not adequately improved, while physicians may not be inclined to initiate or intensify treatment [10,11]. The newly developed Psoriatic Arthritis Impact of Disease Questionnaire (PsAID) is based on patient insights and shows high consistency with individual patient-reported outcome measures [12,13]. Although physicians are not always inclined to target all domains when evaluating responses and tailoring therapy, the majority of patients are concerned with intractable symptoms that inhibit their appearance and ability for daily work and leisure. 

Pain, fatigue, and skin problems rank among the most important patient concerns [12]. High correlations of PsAID with fatigue may indicate that this particular manifestation is a significant constituent in the patient view on PsA [12]. High levels of fatigue are common in PsA, which can be linked to both disease and patient-related factors [14]. Fatigue can be driven by pain, disability, and psychological distress, while changes in pain and depressive symptoms show a degree of consistency under longitudinal evaluation [15,16]. Studies have described how enhanced pain, fatigue, physical impairment, sleep disturbances, and anxiety/depression are mutually intertwined [17]. The relationship between symptoms is multifaceted and likely cannot be attributed to underlying inflammatory factors alone. Data indicate that improvements in fatigue associated with biologic therapy are correlated with reduced joint pain, while ameliorating depressive symptoms are not strictly tied to skin nor joint improvements [18]. High ranking of psoriatic lesions exemplifies how potential social stigmatization or individual discomfort (due to pain and itchiness) is highly detrimental to the patient’s perceptions of the disease. Some studies have suggested that while both severity and stigmatization impact skin-related QoL, mental health is mostly influenced by stigmatization [19]. Cognitive and behavioral aspects provide a potential link between chronic pain, disturbed sleep, and depression [20]. There is also a complex relationship of pain with mood disturbances [21] and disability [22] that we do not fully understand. It is beneficial to think of these factors as an interconnected network that can mutually interact and exacerbate each constituent. Mood and sleep disorders, fatigue, and employment status are all independently related to QoL in PsA [23]. PsA impacts emotional, social, and work-related aspects of life even when treated [24], which shows that improving one domain of disease (i.e., inflammatory activity) does not immediately yield benefits in others. PsA shares a different pattern of QoL impairment from other inflammatory arthritides [25]. Another factor adding to the complexity in disease manifestations and psychosocial impact are cross-cultural differences with regard to social and emotional impact [26] that emphasize a comprehensive biopsychosocial approach is needed. 

Cross-sectional data gathered in nine countries indicate 40% of patients with PsA who are currently treated with disease-modifying antirheumatic drugs (DMARDs) (majority considered as mild severity) experience anxiety or depression tied to PsA. The majority were inclined to the view that both joint and skin symptoms are responsible [27]. PsA patients also have higher rates of subjective health complaints than psoriatic patients, to which both somatic (increased neural sensitivity to stimuli) and cognitive (greater focus on disease) sensitization are thought to contribute [28]. Helplessness and worrying with low acceptance are common in patients with a negative illness perception, while treatment does not benefit the majority of patients who initially suffer from psychological distress [29]. Composite disease activity indices and pain are reportedly higher among patients with anxiety and PsA, which can also lead to an impact on QoL [30]. Compared to the general population, the odds of receiving a diagnosis of behavioral and mental disorders are higher after PsA diagnosis [8]. Fears over future, poor coping, and negative emotions of fear and frustration have been described in PsA [31]. Antidepressant use is also not common [32,33]. Patients may be inclined to conceal suicidal ideation or resort to harmful behavior (e.g., alcohol misuse) [31]. Discussing the patients’ views, emotions, and behavior can allow for identification of “red flags” that may be responsible for psychological distress. One in three patients with PsA who are screened for anxiety will experience symptoms of at least mild severity, with one in five patients showing signs of at least mild depression. The prevalence figures for moderate disease amount to 21 and 14% for anxiety and depression, respectively [34]. Comparative studies show higher rates of depression in PsA than in rheumatoid arthritis (RA) [35] and psoriasis [33]. Emotional processes can drive behavioral (dysfunctional coping, low physical activity, and social interaction), cognitive, and physiological pathways that lead to patients experiencing greater sensitivity and intensity of pain as well as physical disability [36]. Pain has been associated with catastrophizing and reduced coping [37], which underscores the importance of multidisciplinary care to achieve benefits in several patient-oriented domains. Emotional and cognitive responses are subject to longitudinal change and argue against fixed personality traits [22], which indicates they can be targeted by intervention, but can also deteriorate if the disease exacerbates or external factors come into play. Follow-up on psychosocial issues is very important. Studies have examined how coping strategies change after multidisciplinary pain treatment. With depressive symptoms and disability worsening over follow-up, coping strategies changed from adaptive to maladaptive. Maladaptive responses, catastrophizing, and negative beliefs over being disabled impact perceived disability and depression [38]. Changing illness perception and coping to the unpredictable and chronic nature of PsA may reduce symptom severity and encourage patients to undertake a candid and pro-active treatment stance. 

Nearly 65% of patients with PsA are concerned with side effects of long-term medication, with 90% expressing a belief that improved therapies are necessary [11]. Over 40% patients suffering from immune-mediated inflammatory diseases report ambivalence towards their medication, with young and concerned (over drug safety and necessity) patients exhibiting worse adherence [39]. Cognitive factors such as patient beliefs over medication, aside from negative mood or low social support have been shown to impact adherence [40,41]. Comprehensive reviews have identified lower perception of treatment knowledge and patient-provider discordance as associated with non-adherence. Interestingly, the link between depression and non-adherence seems to be more consistent than for anxiety [42]. Promoting a positive representation of illness may favor coping strategies that will reduce distress and improve adherence [43]. Follow-up data based on Danish health registry claims compiled from over 1750 PsA patients indicate depression and anxiety were markedly more prevalent in the group with higher comorbidity scores. In longitudinal analyses, patients with lower comorbidity scores and absence of depression and anxiety have better treatment persistence. In terms of therapy efficacy, optimal responses are less frequently observed in subjects with higher comorbidity scores [44]. These findings illustrate that approaching an individual with a suboptimal drug persistence or response may require a more comprehensive appraisal of comorbidity and psychological status rather than attribution to one causal factor.

Pain is a major complaint in a variety of rheumatic musculoskeletal conditions [45] that can impair physically and carries a negative socioeconomic and psychological burden [46]. It represents a core outcome for trial and observational studies [47]. In PsA, bodily pain is more common than in RA [48] and 20–30% of patients may experience widespread pain (without fibromyalgia) [49] or neuropathic pain [45,50,51]. Even among patients receiving biological agents, pain remains a common finding that is tied to impaired physical function, QoL, and productivity measures [52]. Centrally driven conditions (mood, fatigue, sleep disturbance) may be regarded as an aspect of central pain, which reflects abnormal central nervous system function [46]. Widespread pain has been tied to less satisfactory patient and composite scores, as well as therapy outcomes [49]. A proportion of patients with PsA experience neuropathic pain, which is thought to reflect mechanisms of abnormal pain processing and central sensitization [53]. PsA patients experience lower pain thresholds [54]. Neuropathic pain is characterized by suboptimal response to drug therapies and shares a relationship with psychosocial factors, including comorbid depression and anxiety [55]. 

One of the prevailing theories considers fibromyalgia (FM) as the extreme spectrum of central sensitization [49]. In one study evaluating neuropathic pain features among PsA patients, comorbid FM syndrome was observed to be the only independent predictor [51]. Diffuse pain is a hallmark of FM that can make the differential and treatment decisions in PsA problematic. Systematic reviews indicate comorbid FM is common in chronic inflammatory arthritis and can amplify disease activity scores [56]. Claim-based studies estimate FM prevalence in PsA at approximately 17% [57]. Current diagnostic measures allow for differentiation between inflammatory arthritis and FM, but are limited in other aspects (e.g., failure to capture central sensitization) [58]. It has been shown that psychological distress, self-efficacy, and physical function are important determinants of QoL in FM, which points to the importance of a multidisciplinary approach among these patients [59]. One of the caveats when stating diagnoses also relates to the differential with functional syndromes or somatization disorders. Rheumatic complaints can lead to attribution to somatic symptom disorders by physicians. FM is tied to distressing somatic symptoms of a life-impairing nature, but it is difficult to evaluate whether their character is disproportionate or excessive. It has been argued that this may affect the reliability of conventional DSM-5 criteria in this population [60]. 

## 3. Emerging Concepts: Inflammation and Disease Activity

The impact of disease is multidimensional and treatment success should also be evaluated with regard to patient domains of importance. The IL-23/IL-17 and TNFα axes have been acknowledged with a central role in skin and joint manifestations of psoriasis, which has translated into specific cytokine-targeting agents that show good efficacy in PsA [2,61]. However, while biologic agents and DMARDs are often effective in improving arthritis, the benefits in several intractable, nonspecific symptoms remain variable. Underlying inflammatory mechanisms that underlie arthritis can differ from mechanisms leading to pain, fatigue, and psychological morbidity. Therapy response in PsA with concurrent depression is suboptimal. Intricate relationships between depression, pain, and inflammation have been described [62]. Systematic reviews indicate that DMARDs (TNFα, IL-12/23, IL-17, or phosphodiesterase 4 inhibition) lead to significant, but small improvements in fatigue. In comparison, alleviating pain is more evident, which may be tied to prevailing inflammatory components strongly tied to PsA activity. Fatigue can occur despite effective treatment of rheumatic disease, and models including cognitive and behavioral factors are discussed [63]. These findings indicate the need for comprehensive assessments to inform the physician on modifiable factors responsible for suboptimal therapeutic efficacy. 

Reviews on the efficacy and safety of biologics in psoriasis and PsA have been previously published [64]; therefore, we focus on novel agents that have garnered much attention. The Janus kinase/signal transducers and activators of transcription (JAK-STAT) pathway represents a recent target of therapeutic interest in PsA. Synovial T cells of PsA patients have been compared with peripheral blood of healthy controls, and have been described with increased JAK1/STAT3/STAT1 [65]. Interactions between Th17 and Treg cells within a milieu of IL-1, IL-6, and IL-23 may contribute to arthritis-driving pathways [66]. Synovial fibroblasts from PsA synovium have enhanced phosphoSTAT (pSTAT) 1 and 3 expression, which can be inhibited by tofacitinib, a JAK inhibitor. JAK1/STAT1/STAT3/STAT5 phosphoproteins were also shown to be markedly enhanced in synovial fluid samples, alongside higher IL-6 in peripheral blood [66]. Functional effects have been confirmed by reducing fibroblast migration, invasion, and network formation. Effects were also observed in both pSTAT1 and 3 inhibition with abrogation of pro-inflammatory cytokine secretion (e.g., IL-6, IL-8) in synovial explants [67]. IL-23 has been shown to induce activation of JAK-2 and STAT3, while also upregulating IL-17 in CD4+ memory T cells [68]. Inhibition of tyrosine kinases by tofacitinib hampers signal transduction through STATs and represents a new therapeutic avenue in inhibiting the prominent cytokine axes of PsA.

Mechanisms underlying pain in inflammatory arthritis are being explored with the prior concepts stemming from a byproduct of joint injury extended to neuroinflammatory components. Pain sensitization can be tied to psychological aspects. Pain does not always correspond to the degree of inflammation, and while DMARDs can relieve inflammatory pain, the mechanisms of central regulation that lead to widespread pain can still persist and affect patient evaluation of disease [46,69]. Understanding the pathogenesis of pain, including neuropathic components and the relationship with FM, represents an interest that may facilitate the development of novel therapeutics. In rat models of neuropathic pain, neural damage leads to the activation of JAK/STAT3 in spinal cord microglia with increases in IL-6 concentrations. Both neutralization of IL-6 leads to reduced STAT3 phosphorylation, and intrathecal JAK2 inhibition leads to suppression of STAT3 [70]. The ability to integrate and mediate both pro- and antinociceptive activity has been described for the JAK/STAT3 pathway. This may relate to the activation of different gene transcription pools and shift microglial polarization to either terminate inflammation (M2) or promote pronociceptive factors [71]. Evaluating the potential benefits of JAK inhibitors in improving pain is also compelling.

The shift from the monoaminergic theory of depression to recognizing the importance of cytokine signaling, neuroinflammatory, and neuroendocrine pathways with external stressors creates a complex and multifaceted model of depression [62,72,73]. Imaging studies have tied peripheral inflammation to altered brain structure integrity in depression [74]. Biological components seem to be influenced by psychological and socioenvironmental stressors that impact immune and endocrine responses, leading to altered glucocorticoid sensitivity and excess inflammation, particularly if chronically upheld. The concept of pro-inflammatory shifts in immune populations, based on the perpetual perception of threat, leading to early development of symptoms (hypervigilance, sensitivity to pain, social anxiety), which gradually progress to chronic pain, depressive mood, and precipitate inflammatory, is postulated [75]. Depression shares a close relationship with inflammation in PsA, though the efficacy of disease modifying anti-rheumatic drugs is not optimal [62]. Data from RA suggests that the presence of depressive symptoms reduces the likelihood of attaining good responses and controlling disease over time [76]. A systematic appraisal of evidence indicates adalimumab, etanercept, and ustekinumab reduce depressive symptoms in moderate-to-severe psoriasis [77]. Indirect effects based on reduction in anti-depressants use may also be tied to biologic therapy in pooled studies of psoriasis and psoriatic arthritis, though a lack of comparative population with systemic treatment limits the generalizability of these findings [78]. These data imply that comorbid depression can confer a more difficult-to-treat risk factor in PsA. Prospective cohorts have shown that baseline mental health conditions can reduce likelihood of remission in RA and potentially PsA [79]. 

Greater understanding of inflammatory components in depression could pave the way for personalized therapy. Meta-analyses of clinical trials in inflammatory diseases show cytokine targeting leads to modest improvements in depressive symptoms, specifically if they are elevated at the baseline. Notably, the effect for IL-12/23 inhibition is substantial (95% CI 0.26, 0.70), even after correction for physical health outcomes [80]. Longitudinal data indicate biologics decrease the incidence of depressive symptoms in psoriasis cohorts [81]. Elevated TNFα in depression may be responsible for lesser efficacy of conventional antidepressants [82], which would suggest future tailoring therapy to individual cytokine profiles. However, systemic concentrations may not correspond to tissue levels and can be subject to other interfering factors. Data for PsA are scarce as comprehensive reviews examining depression and anxiety in PsA have found only one study that met inclusion criteria to explore treatment effects and reflects a high risk of bias that indicates quality data are necessary [83]. It has been noticed that although cytokines involved in PsA (IL-6, IL-17, TNFα) can be elevated in depression or anxiety, conflicting data indicate no apparent link between depression and circulating IL-23 or IL-17 [62]. Meta-analyses in depression have indicated responders to antidepressants have a unique cytokine profile and specific shifts can be tied to therapy success [82]. Differentiating systemic and psychological symptoms as a byproduct of enhanced inflammatory pathways hold potential to stratify patients at greatest benefit of cytokine-targeted therapy (e.g., anti-inflammatory target therapy or anti-depressant choices based on Th1/Th2 cytokine response shifts) [84]. However, without mechanistic understanding, these speculations remain tentative and we are unable to explain whether singular or distinct disease pathways are responsible for specific clinical manifestations. 

Understanding the patient’s perception of disease and provider disparity in defining treatment goals and success remains crucial in facilitating shared decision-making. Discordance in patient and physician global assessments has been shown to indicate a greater burden of the disease from a patient’s perspective. The determinants of these scores were observed in domains of coping, social participation, and fatigue, which point to disagreement in psychological, but not physical dimensions [85]. This is clinically important as the evolution of symptoms is often mutually intertwined. Studies have suggested that fatigue, pain, and poor mental health predispose to underrepresentation of disease activity, while reliance on swollen joint counts may lead to the contrary [86]. Moreover, remission defined across a variety of composite disease activity indices and patient-reported outcomes can differ and will not always be consistent with more objective surrogates of inflammation. Neuropathic-like pain in PsA is more commonly associated with higher disease activity assessments [53]. Studies report higher neuropathic pain scores and lower swollen-to-tender joint count ratios in PsA than in other spondyloarthropathies or RA. The relationship between pain with patient-reported outcomes and composite indices has also been observed, though some objective surrogate measures of inflammation do not share this association. These findings imply that for a proportion of patients, the intensity and character of pain may occlude the evaluation of PsA activity [50]. Iannone et al. examined 238 patients with PsA, of which 58 also suffered from FM. Aside from a higher frequency of polyarticular disease and increased BMI, both first and second line biologic treatment retention and the rates of achieving remission and low disease activity were markedly worse [87]. Composite disease activity scores have been observed to be markedly higher in observational studies of FM and PsA. Moreover, the likelihood of achieving minimal disease activity was substantially smaller in the group with FM, with none of the examined patients meeting the criteria. Interestingly, more objective measures such as C-reactive protein and swollen joint counts did not differ across groups [88]. It has been proposed that the most pressing concern over comorbid FM is the misleading of both patients and providers in evaluating disease activity, which may lead to overtreatment if tight control strategies are undertaken [87,89]. 

## 4. Treatment Modalities

Due to the heterogeneity of disease, the expert stance regarding diagnosis and evaluation requires a multidisciplinary approach with regard to comorbidity. Improving QoL and psychosocial aspects, while treating and modifying risk factors is necessary alongside appropriate interventions to control inflammatory activity early [3]. It has been shown that tight cooperation between rheumatology and dermatology specialists can be structured to facilitate optimal management of PsA based on disease control and improvement in QoL [90]. Although data are still accumulating, systematic reviews in psoriasis and PsA indicate that benefits in multidisciplinary consultation extend to substantially improve patient satisfaction alongside clinical outcomes [91].

Conventional and biologic DMARDs are the staple of the rheumatologist’s treatment armamentarium with their efficacy and safety in inflammatory arthritis. New agents targeting different cytokine signaling pathways hold promise to improve on the current efforts to control disease manifestations with preliminary data indicating that benefits in patient-oriented outcomes are favorable [2,64]. Controlling disease activity and improving functional status and pain can be achieved with baricitinib and methotrexate when compared to a combination with adalimumab in RA. JAK inhibitors may alleviate pain more rapidly and among a greater proportion of patients. Novel drug targets within JAK-STAT-signaling pathways may affect pronociceptive and inflammatory mechanisms to a greater extent than current agents [92,93]. On the other hand, if pain results from both cognitive, behavioral, and inflammatory components, a conjunction of different pharmacologic and psychotherapeutic treatment modalities can be useful. Despite common agreement of its relevance, psychological support is considered lacking among rheumatology centers. Psychologists are still rare among multidisciplinary teams in rheumatology, while referral does not always seem effective under current healthcare organization. Clinicians themselves often do not have time or relevant training to provide different modalities of psychotherapy [94]. Moreover, the synergistic effect of DMARDs and antidepressants remains an open question for investigation and it has been noted that placebo effects on depression and anxiety have to be scrutinized with regard to the relative benefits attributed to antidepressants [62]. Bridging the divide between psychotherapy and management of difficult manifestations of rheumatic disease is a unique opportunity to improve patient satisfaction and translate multidisciplinary care into better clinical outcomes. Cost-efficacy of psychotherapy could reduce strain on providers and healthcare resource utilization, particularly if combined with digitalized tools that may improve convenience and accessibility [95,96]. 

Studies indicate that the incidence of depression and anxiety is higher in inflammatory diseases prior to diagnosis when compared with the general population [97]. Patients treated with biologic DMARDs or other conventional agents have reduced prescription rates for antidepressants and hypnotics, which may suggest that controlling inflammation provides a sufficient improvement in a proportion of patients such that further pharmacotherapy is not warranted [98]. We are inclined to the view that causal attribution of affective disorders in PsA to one factor is improbable and care should be multidisciplinary. While in among some patients active systemic inflammation (marked by surrogate measures such as SJC, acute-phase reactants) is likely strongly tied to psychological distress, in others, the nature of mood disturbance with maladaptive coping, catastrophizing, and negative socioenvironmental aspects can represent a dominant factor that is modifiable. 

Considering the inflammatory component in depression, as well as psychosocial factors that can influence immunity, increased awareness of psychotherapy should be promoted. Comprehensive reviews have shown that cognitive-behavioral therapy (CBT) is associated with surrogate measures of positive immune function: A reduction in pro-inflammatory cytokines, higher immune cell counts, natural killer cell activity, and immune outcomes [99]. CBT is a well-established intervention with long-term effects for a variety of psychiatric disorders such as major depression, generalized and social anxiety, or post-traumatic stress disorders [100]. In chronic pain conditions, systematic reviews have indicated a variety of small to moderate benefits in improving disability, catastrophizing, mood, and pain, though the significance is relevant to the study comparator (active treatment or treatment as usual) [101]. In psoriasis, a recent comprehensive appraisal of evidence showed CBT-based psychotherapy is effective, while others are not. Efficacy has been justified by reduction of stigmatization, correction of misconceptions, and improving understanding of disease. Additionally, the patient may be encouraged to undertake a more active role. Interestingly, with greater severity of the disease, the efficacy may be enhanced [102]. From a psychological background, patients with functional somatic syndromes will tend to display higher levels of neuroticism, which relates to poor coping strategies (i.e., symptom catastrophizing), which in turn may lead to impaired social function and poor physical health. These patients may be at particular benefit of cognitive behavioral therapy (CBT) [103]. Reducing symptoms of pain and catastrophizing may also be achieved through CBT based on reports from FM [104].

Electronic CBT approaches have been demonstrated to improve psychological distress in RA, which can also extend to anxiety and quality of life, as seen in psoriasis [105,106]. Participants in programs with CBT interventions demonstrate significant improvements in sleep continuity and quality, alongside a relatively durable response time [107]. These modalities are particularly relevant for a stepped care model. Other supportive measures may include mindfulness-based approaches that can improve coping mechanisms, which can jointly improve emotional regulation (mindfulness) and encourage cognitive control [108]. A randomized controlled trial in RA has shown a benefit of 10-session mindfulness interventions in self-efficacy, emotion processing, fatigue, and psychological distress [109]. Interventions to improve patient knowledge (high quality educational materials) show improved understanding, coherence, and perception of control without elevating anxiety [110]. In chronic musculoskeletal pain, the addition of educational interventions can improve self-efficacy, and reduce pain intensity and interference [111]. These findings suggest that education and promotion of positive coping strategies, which support the patient’s belief over their own influence on PsA symptoms, can be beneficial, though remain transient and require maintenance care (e.g., more frequent clinician follow-up, support groups, structured psychotherapy). Little is known about the impact of psychotherapy in PsA, which marks an important theme for the research agenda.

## 5. Outlook and Future Directions

Patients with PsA suffer from a chronic inflammatory disease that leads to joint and skin problems with intractable fatigue and pain, with negative psychosocial impact that contributes to a significantly reduce quality of life. Diagnostic delays with clinical inertia and misattribution of symptoms can frequently occur and augment mental distress. Maladaptive coping mechanisms can develop. Symptoms in PsA may be interfaced by inflammation but exacerbated by poor cognitive and behavioral responses. As our understanding of pathogenesis develops, the relationships between inflammation, pain, fatigue, and mental health in PsA become intertwined. JAK inhibitors are novel agents that control crucial cytokine signals involved in pathogenesis and may provide benefits in controlling disease activity and pain. When evaluating disease activity, physicians should utilize outcome measures developed by both specialists and patients that best reflect the multidimensional impact of PsA. Assessments and treatment should be aided by cooperation between specialists. Creating multidisciplinary teams and potentially structuring the cooperation process is a broad scope view on how to improve patient outcomes. Psychotherapy modalities represent a new avenue to improve psychosocial domains of PsA. Quality data on the efficacy of antidepressants and disease modifying drugs are necessary to assess their impact on depression and anxiety in PsA. Promoting a positive representation of illness, encouraging adherence, and developing strategies to combat pain and fatigue represent new areas of clinical interest.
